# Malaria and Geohelminthiasis Coinfections in Expectant Women: Effect on Maternal Health and Birth Outcomes in a Malaria Endemic Region in Kenya

**DOI:** 10.1155/2018/2613484

**Published:** 2018-11-29

**Authors:** Wekesa Antony Wanyonyi, Chrispinus Siteti Mulambalah, David H. Mulama, Elizabeth Omukunda, Darwin Injete Siteti

**Affiliations:** ^1^Department of Biological Sciences, Masinde Muliro University of Science and Technology, P.O. Box 190, Kakamega, Kenya; ^2^Department of Medical Microbiology & Parasitology, College of Health Sciences, Moi University, P.O. Box 4606, Eldoret, Kenya; ^3^Department of Clinical Medicine, Jomo Kenyatta University of Agriculture and Technology, P.O. Box 62000, Nairobi, Kenya

## Abstract

Geohelminthiasis and malaria coinfections in pregnancy are common in sub-Saharan Africa. The consequences of the disease combination on maternal health and birth outcomes are poorly understood. For a better understanding of this coinfection in expectant mothers, a cross-sectional study was carried out to evaluate the effect of the coinfection on maternal health and birth outcomes in expectant mothers in Bungoma County, Kenya. To collect data on malaria and maternal haematological parameters, blood samples were obtained from 750 participants aged 18-49 years and analyzed. Haemoglobin and eosinophils levels were determined by coulter counter while malaria parasitemia levels and red blood cell morphology were assessed by preparing and observing blood smears under the microscope. Fresh stool samples were collected and processed for identification and quantification of geohelminths species using Kato-Katz. Harada Mori technique was used to increase chances of detecting hookworms and* Strongyloides *infections. Neonate's health was evaluated based on the appearance, pulse, grimace, activity, and respiration (APGAR) scale. Parasites identified were* Plasmodium falciparum, P. malariae, P. ovale, Ascaris lumbricoides, Necator americanus, Enterobius vermicularis, *and* Schistosoma mansoni*. The prevalence of geohelminths, malaria parasites, and coinfection was 24.7%, 21.6%, and 6.8%, respectively. Those coinfected with geohelminths and malaria parasites were four times likely to have anaemia (OR 4.137; 95% CI 2.088-8.195; P=0.001) compared with those infected with geohelminths or malaria parasites alone (OR 0.505; 95% CI 0.360-0.709; P=0.001 and OR 0.274; 95%CI 0.187-0.402 P=0.001, respectively). The odds of having preterm deliveries (OR 6.896; 95% CI 1.755-27.101; P=0.006) and still births (OR 3.701; 95% CI 1.008-13.579 P< 0.048) were greater in those coinfected than in those infected with either geohelminths or malaria parasites. Geohelminths and malaria coinfections were prevalent among study participants; consequently the risk of maternal anaemia, preterm deliveries, and still births were high. Routine screening and prompt treatment during antenatal visits should be encouraged to mitigate the adverse consequences associated with the coinfections.

## 1. Background

Parasitic infections such as malaria and geohelminthiasis are life-threatening as well as the leading cause of mortality in developing countries particularly in risky groups, such as children under five years and pregnant women [1]. Expectant mothers are more vulnerable to infections because, during pregnancy, there is a transient depression of cell-mediated immunity that allows foetal allograft retention which on the other hand interferes with resistance to various infectious diseases [2]. Immunological factors influence rates of coinfection because helminths modulate host immune responses both to themselves and to concurrent infections. That is why pregnant women, immunologically compromised, are highly susceptible to parasitic infections such as malaria and soil-transmitted helminthiasis [3]. Immunological studies show that the impact of geohelminths and malaria parasites is intensified when they coexist [4, 5].

A number of inconsistencies regarding the relationship between geohelminthiasis and malaria coinfection in pregnancy abound in literature. In a study undertaken in Nigeria, it was demonstrated that over 45% of* Plasmodium*-infected pregnant women also harbored various geohelminths [6]. A contrary study finding indicated no association between geohelminths and malaria parasites in pregnancy [7].

Infection with geohelminths particularly hookworms and malaria parasites is associated with decreased haemoglobin levels which lead to anaemia. These may have disastrous consequences in pregnancy [8, 9]. Furthermore, coinfection involving hookworms and* Plasmodium falciparum* has been reported to have additive impacts on haemoglobin by increasing the susceptibility to* P. falciparum* which have greater consequences on pregnancy outcome, because it results in intrauterine growth retardation, low birth weight, preterm delivery, and high neonate mortality [10].

A study done in Ghana found that anaemia in pregnant women, coinfected with geohelminths and malaria parasites, was associated with low birth weight, preterm delivery, and small birth weight for gestational age. Most of these adverse effects appeared to be driven by malaria parasite infection. The only significant effect of geohelminths alone was an increase in the risk of being small for gestational age [11].

In another related study, women coinfected with geohelminths and malaria had higher birth weights [12]. These inconsistencies necessitated further studies for a better understanding of these coinfections in pregnancy [13]. A hospital based study was carried out to determine the effect of geohelminths and malaria parasites coinfection on maternal health and birth outcomes among pregnant women residing in a malaria endemic region in Kenya.

## 2. Methods

### 2.1. Study Design, Site, and Setting

A quantitative cross-sectional hospital based study was carried out from March 2016 to January 2017. Consecutive sampling was used to recruit 750 expectant mothers presenting for antenatal services. The study was carried out at Bungoma Country Referral Hospital (BCRH), located between latitude 0° 34′0”N and Longitude 34° 34′ 0”E in Bungoma County, Kenya. The County is situated in a malaria endemic zone with perennial disease outbreaks. It has an estimated human population of 1,375,063 with an area of 3,032.2sq km, according to Kenya 2009, national census [14]. The County comprises 9 subcounties, namely, Kanduyi, Bumula, Sirisia, Kabuchai, Kimilili, Webuye East, Webuye West, Tongaren, and Mount Elgon. It has a tropical climate characterized by hot and humid conditions and two rainy seasons: the long rains (April to August) and the short rains (October to December). The predominant ethnic group is Luhyas (Bukusu), Teso, and Sabot, who practice subsistence farming. The referral hospital provides antenatal and delivery services to slum, rural, and urban communities.

### 2.2. Sample Size Determination

According to guidelines put forth by the World Health Organization (WHO), a sample size of 250 complying individuals in a geographically distinct community is needed to assess the prevalence and intensity of soil-transmitted helminth infections in each community [15, 16]. The study area covered three community units and an overall sample size of 750 pregnant women was considered appropriate.

## 3. Data Collection

### 3.1. Sampling Frame and Inclusion and Exclusion Criteria

Pregnant women seeking for delivery services at the Bungoma County Referral Hospital, aged between 18 and 49 years, who had lived in Bungoma County for the last 6 months and willing to consent were recruited in the study.

Expectant mothers, who were nonresidents, were below 18 and above 49 years of age, and those not willing to give consent and those on antimalarials or anthelmintics or haematinics were excluded from the study.

### 3.2. Stool Sample Processing and Geohelminths Identification

All consenting pregnant women were provided with labeled screw caped stool container plus a scoop and informed on how to transfer about 5 grams of fresh stool sample into a container. Each container was identified by the code number of the participant. Stool sample was collected from each participant during antenatal clinic (ANC) visit.

Kato-Katz thick smear technique was used to process stool, identify and quantify the number of geohelminths eggs per gram of faeces [17]. Each stool specimen was prepared by passing through a sieve to remove debris. Two slides for readers A and B were labeled. Filtered stool sample was transferred into a hole in a template mounted on each glass slide calibrated to hold 47.1mg of stool. Each preparation was covered with cellophane soaked in glycerin and malachite green. The preparations were then turned upside down on a flat surface and pressed gently to spread the stool sample evenly. Each slide was read by a separate qualified laboratory technologist to increase the sensitivity of geohelminths detection and verification of negative slides. The readings were done microscopically using ×10 and ×40 and average number of eggs in the two slides calculated. To ensure quality results, the slides were examined within one hour after preparation to avoid over clearing of hookworm eggs by glycerin. Specific helminth eggs and larvae were identified morphologically, counted, and recorded. As a quality control measure, 10% of already examined stool slides were reexamined by the principal investigator.

Haratamori technique [18] was used to culture and hatch hookworm eggs to differentiate* Necator americanus* filariform larvae from those of* Ancylostoma duodenale*. The procedure involved the use of filter paper strips of about 5 inches slightly tapered at one end for each stool specimen confirmed to contain hookworm eggs. One gram of each faecal sample was smeared at the centre of the strip. Four millilitres of distilled water was added to 15 millilitre conical centrifuge tube. The paper strips were inserted into the tube such that the tapered end was near the bottom of the tube with water level slightly below the faecal point. The tube was plugged using cotton wool and allowed to stand upright in a rack at 25°C for 10 days. Small amount of the fluid was withdrawn from the bottom of the tube and a smear was prepared on a glass slide. The preparation was cover slipped and examined microscopically using 10 x objective. Only* Necator americanus* filariform larvae were recovered from stool specimens. Filariform larvae of* Necator americanus* were identified based on their morphological features: average larval length 59*μm*, pointed head and tail, oesophagus with thistle funnel shape (oesophageal bulb), presence of gap between oesophagus and intestine, and oesophagus length approximately 1/3 in relation to entire body length [18].

### 3.3. Blood Collection and Processing for Malaria Parasite Identification

Maternal peripheral blood was collected during antenatal clinic (ANC) visits. Cubital vein of each participants arm was sterilized using 70 % methylated spirit and 5 millilitres of blood was collected using a sterile syringe and needle. The blood was then transferred into a cryovial containing anticoagulant ethylenediaminetetraacetic acid (EDTA).

For malaria parasites examination, two drops of blood was put on clean glass slide to prepare thick film and thin blood film and then air-dried in a horizontal position. Thick blood film concentrates malaria parasites for easier viewing while thin blood film facilitates malaria species identification by their morphological features. Thin blood films were fixed in 70% methanol for 30 seconds and air-dried. Both thin and thick films were stained using 10% Giemsa for 10 minutes. All asexual forms of malaria parasites (trophozoites and schizonts) in each preparation were identified microscopically using oil immersion objective ×100 and recorded. Malaria parasites density per microlitre of blood was calculated by counting the number of malaria parasites against 200 white blood cells (WBCs) and multiplying by 8,000 to get malaria parasites/*μ*l of blood [19]. At least 100 high-power fields were examined before a film was declared negative. For quality control 10% buffered (pH 7.2) Giemsa stain was prepared for use after every 6 hours. Known malaria positive controls with low parasitemia were stained and examined daily to check the quality of the stain. 10% of read blood smears were reexamined for quality control. Part of the blood sample was used to assess haemoglobin and eosinophil levels and red blood cell (RBC) morphology.

### 3.4. Estimation of Haemoglobin and Eosinophil Levels

Maternal haemoglobin levels were measured using a portable haemoglobinometer (Hemocue Lee Diagnostic Inc. Switzerland) system by putting 2 microlitres (ul) of blood in a microcuvette preloaded with stabilising reagents. The microcuvette was then immediately placed into a portable spectrophotometric instrument and the digital reading of the haemoglobin concentration of each subject was read within 10-20 seconds. Control blood samples with known values of low haemoglobin levels (Hb<11.0g/dl) and high haemoglobin values (Hb 18g/dl) were run daily to validate the accuracy and reliability of the haemoglobinometer results

Coulter counter machine CBC5 (Coulter corporation Miami, FL, USA) was used to evaluate eosinophil levels. A cryovial containing 5mls of blood was placed in the coulter machine which sacks 10*μ*l of blood following specific command and each subject result was read within 10–20 seconds. To diagnose iron deficiency (presence of hypochromic and microcytic red blood cells) peripheral blood films (pbf) were made from already collected blood, air-dried, and fixed using 70% methanol. Staining was done using 10% Leishman stain for 10 minutes and examined using oil immersion with ×100 objective [19]. Microcytic hypochromic red cells were identified and recorded based on their small size and pale staining characteristics. Daily running of known control blood samples to validate accuracy of the Coulter counter and haemoglobinometer machine before running test samples was done. Weekly running of quality assurance (QA) to check the effectiveness of quality systems that are in place, for example, proper recording of the results, was also done.

### 3.5. Assessment of Neonatal Outcomes

Data for neonatal outcomes were obtained, recorded, and evaluated. Gestation period was assessed by palpation before delivery. After delivery, neonatal conditions (alive or stillbirth) were assessed, weight was measured to the nearest 0.1kg using an electronic balance, and appearance, pulse, grimace, activity, and respiration (APGAR) scale commonly were used to assess neonates health after birth. Based on the APGAR scale, the neonate's condition at birth was scored as normal, high chances of survival, low chances of survival, or dead.

### 3.6. Data Analysis

*Data Analysis Was Performed Using SPSS Version16. *Descriptive statistics were used to summarize data and t-test was used to compare means. The relationship between geohelminths and malaria coinfection and different explanatory variables was done using chi-squire (*X*^2^) test. Multiple logistic regressions were used to determine the effect of malaria and helminth coinfection on maternal anaemia and birth outcomes. Statistically significant variables in bivariate analysis were entered into a model, odds ratios and 95% confidence intervals (CI) were calculated, and P value was set as t≤ 0.05. Models were run for each adverse outcome separately, adjusting for maternal anaemia.

### 3.7. Ethical Considerations

The study was approved by Masinde Muliro University of Science and Technology Institutional Review Board (approval number MMU/COR403OO9 (57). Further approval was obtained from Bungoma County Referral Hospital. Oral and written informed consent was obtained from all study participants in any of the languages (English, Kiswahili). Pregnant women were explained to the study objectives and procedures. They were requested to sign written individual consent form. Study participants were given the option to withdraw from the study at any time they wished. Data was coded and kept strictly confidential. Information of the results was shared with study participants. Those infected with geohelminths and or malaria parasites were treated with antimalarial, antihelminthic, and haematinic drugs free of charge at the hospital in accordance with clinical guidelines of WHO and Ministry of Health, Kenya.

## 4. Results

### 4.1. Characteristics of Pregnant Women and Their New-Borns

A total of 750 pregnant women were recruited in the study with gestation mean age of 24.63 ± 5.22 years. Majority of the participants (71.1%) were in the age range 18–27 years. The overall participant's characteristics are presented in Table 1.

### 4.2. Prevalence of Geohelminths and Malaria Parasites in Pregnancy

The occurrence of geohelminths, malaria parasites, and coinfection in this study was 24.7%, 21.6%, and 6.8%, respectively. The geohelminths identified were* Ascaris lumbricoides, Necator americanus*,* Enterobius vermicularis,* and* Schistosoma mansoni*. Malaria parasites identified were* Plasmodium falciparum*,* Plasmodium malariae,* and* Plasmodium ovale*.

### 4.3. Risk Factors for Maternal Anaemia

Although the mean haemoglobin level was 11.62 g/dl, 95% CI (11.49-11.74), a total of 367(48.9%) of expectant mothers were anaemic. Coinfection of* Necator americanus *and* P. falciparum* was associated with maternal anaemia** (***χ*^2^ 10.288, p=0.006). Majority of pregnant mothers coinfected with* Necator americanus* and* P. falciparum *had microcytic hypochromic red blood cells 24(47.1%), (*χ*^2^ 24.866, p=0.001).* Ascaris lumbricoides *and* P. falciparum *coinfection was not associated with maternal anaemia (*χ*^2^ 1.680, p=0.891). A total of 160(21. %) expectant mothers had eosinophilia. Although coinfection between* Ascaris lumbricoides *and* P. falciparum* showed the highest number of eosinophilia 25(51%), the association was not statistically significant (*χ*2 0.943, p=0.967).

Mothers who were infected with geohelminths alone were 4 times likely to have microcytic hypochromic red blood cells an indicator of iron deficiency anaemia (Table 2).

Expectant mothers who had malaria parasites alone were 3-fold likely to have low haemoglobin level (anaemia) Table 3.

Expectant mothers coinfected with geohelminths and malaria parasites were 4- and 5-fold likely to have low haemoglobin level and microcytic hypochromic red blood cells (anaemia) compared to those infected with either geohelminths or malaria parasites (Table 4).

### 4.4. Risk Factors for Adverse Neonatal Outcomes in Single Infections

Out of 185 pregnant women infected with geohelminths alone, 18 (9.7%) had preterm deliveries, 7 (3.8%) abortions, 11 (5.9%) stillbirth (borne dead), and 38 (19.9%) low birth weight and on APGAR score neonates who had low chances of survival were 3 (1.6%). Out of 162 mothers with malaria alone, 26 (16%) had preterm deliveries, 5 (3%) abortions, 8 (4.9%) stillbirth, and 44 (26.9%) low birth weight. On APGAR scale, neonates who had low chances of survival were 7 (4.3%). Mean birth weight for live neonates was 1.92 kg at birth. Chi-square test was used to analyze risk factors associated with adverse neonatal outcomes in single infections and the results presented in Table 5.

### 4.5. Risk Factors for Adverse Neonatal Outcomes in Coinfections

Expectant women coinfected with* Necator americanus* and* Plasmodium* parasites were more likely to experience an adverse birth outcome compared to noninfected women.* Necator americanus* and* Plasmodium* coinfection were associated with stillbirth, P value 0.048 OR 3.701, (1.008-13.579), preterm deliveries p value 0.006 OR 6.896 (1.755-.27.105), low birth weight p value 0.001 OR 9.820 (7.630-12.640), and, on APGAR scale, neonates with low chances of survival, P value 0.001 OR 5.310 (1.860-15.162). Logistic regression was used to analyze the risk factors associated with adverse neonatal outcomes in coinfections and results presented in Table 6.

## 5. Discussion

Poly-parasitism is a public health problem in sub-Saharan Africa. Malaria parasites and geohelminths coinfection are currently receiving a lot of attention because of the likelihood of the complications arising from their coinfections. The observed occurrence of geohelminths and malaria parasites in this study is an indication of moderate transmission and coendemicity of these parasites in the study area.

Our study findings showed that coinfected mothers were at 4-fold increased risk of anaemia as compared to mothers who had either geohelminths or malaria parasites alone. These results are in agreement with a study in Ghana which revealed that coinfection between geohelminthiasis and malaria substantially increased the risk of anaemia in pregnancy [11]. The findings also collaborate with the work of Francis and others [20] who reported a high occurrence of anaemia in mothers coinfected with hookworms and* P. falciparum*. An explanation for this observation could be attributed to the additive effects in the pathogenic mechanisms of the individual the parasites species involved in total haemoglobin concentration. Adult hookworms produce a wide range of protein molecules that facilitate their feeding activities by aiding digestion of intestinal tissues in the process of feeding, while others act as anticoagulants. The anticoagulants ensure a steady blood flow from intestinal ulcers which provides a source of iron, nutrients, and oxygen for the worms. This explains why adult hookworms are associated with unhealing intestinal ulcers and chronic loss of blood and iron in gastrointestinal system. Malaria, on the other hand, is a haematologic disease that causes profound changes in haematological parameters among infected individuals including anaemia which may result from various parasite pathogenic mechanisms. Anaemia a common symptom of malaria may arise as a result of red blood cell (RBC) lysis due to direct parasite invasion, induced intravascular haemolysis, and reduced erythropoiesis in infected patients. Enhanced splenic clearance of infected and modified RBC in malaria patients may exacerbate anaemia. The most widespread and lethal* Plasmodium *species,* P. falciparum,* is consistently associated with bursting and loss of red blood cells during its blood stage, all contributing to haemoglobin loss and anaemia in patients with the coinfections [20].

It was also evident that geohelminths and malaria parasite coinfections were associated with various adverse birth outcomes. Compared to those infected with either geohelminths or malaria parasites, expectant mothers coinfected with geohelminths and malaria parasites were twice likely to give birth to low birth weight babies, 3.7 times likely to have still births, 6.8 times likely to have preterm deliveries, and 5 times likely to have newborn babies with low chances of survival. These findings are comparable to a related study where coinfected expectant women with malaria parasites and geohelminths were 3.0-fold and 2.6-fold at risk of low birth weight and preterm deliveries, respectively, compared with uninfected women [21]. The possible explanation is that adult intestinal worms compete with the host for food and interfere with the absorptive surface. The shared food resources may be insufficient to satisfy the needs of the parasites, the increased demand of the expectant mother, and the growing fetus. Interruption of nutrient acquisition by competition and destruction of absorptive surface in the mother leads to reduced nutrient supply to the fetus and subsequently retarded intrauterine growth and low birth weight. Transplacental transmission is possible during parasite migration in the body and this phenomenon enhances the chances of the parasite infecting fetus before birth. Infected neonates usually have low chances of survival. If not addressed early during pregnancy, this results in retarded foetal growth and low birth weight, stillbirth, and preterm deliveries. Furthermore, RBC infected with* P. falciparum *usually congregate in the placental vascular space. In an active infection in pregnant women, the fetus may be exposed to parasitized maternal RBC. This may induce immunological responses that contribute to early onset of labor and consequently preterm deliveries and still births. These findings highlight the serious adverse health effect of coinfections in pregnancy and the need for early screening and prompt treatment.

Contrary findings have been reported in a study in Nigeria in which no cases of still birth and low birth weight were recorded among pregnant mothers coinfected with malaria and geohelminths [22]. It is not clear whether the study subjects were allowed access to antimalarials, anthelmintics, or hematinics before or during the study. Treatment with these drugs could explain the reported findings because, when used, they modify maternal anaemia and/or birth outcomes. Also, there may have been undetected differences in the status of nutrition and other confounding factors.

A number of other factors are known to induce changes in haematological parameters and birth outcomes during pregnancy. These include, for instance, haemoglobinopathies (sickle cell, thalassemia, and G6PD deficiency), genetics, and environmental factors, nutritional status, and metabolic diseases. The prevalence of haemoglobinopathies is generally very low in any given population and were not reported or observed in any of the participants. During antenatal visits, expectant mothers are advised to keep environment clean at all times, maintain good nutritional status, and get assessed for any metabolic diseases. Therefore, the probability of environmentally or nutritionally induced haematological changes was very minimal and no cases of metabolic diseases were reported among the participants. In this regard, the maternal haematological findings and birth outcomes discussed were attributed to geohelminths and malaria infections under investigation though other factors may have played an insignificant and unobserved role. The potential significance of the coinfections involving geohelminths and malaria in the epidemiology of poly-parasitism is worth exploring in different transmission settings.

## 6. Conclusions

The study demonstrated that expectant mothers coinfected with geohelminths and malaria parasites were at an increased risk of high anaemia and adverse birth outcomes. Geohelminthiasis and malaria were prevalent in Bungoma County. We recommend that screening of these infections be incorporated in the antenatal care program to improve maternal and neonatal health.

## Figures and Tables

**Table 1:** Characteristics of pregnant women and their new-borns.

**Variable**	**Number**	**Percentage**
**Age group**		
18-27	541	71.1
28-37	192	25.6
38-45	17	2.3
**Residence**		
Rural	381	50.8
Slum	220	29.3
Urban	149	19.9
**Employment**		
Employed	158	21.1
Farmer	252	33.6
Business	2	0.3
Not employed	338	45.1

**Table 2:** Association of geohelminths with changes in maternal haematological parameters.

Geohelminths						
Variable	OR	CI	P-value	AOR	CI	P-value
Hb level						
<11.5gm/dl	1.289	1.170-421	0.001	1.106	1.09-1.122	0.001
**Pbf**						
M/h	8.683	5.407-13.943	0.001	4.341	3.569-5.282	0.001
**Eosinophil**						
>6% Raised	0.001	0.000-0.004	0.001	1.465	1.304-1.647	0.001

**∗**95% CI=confidence interval and OR= odds ratio; P<0.05 was considered significant.

**∗**Hb= haemoglobin, Pbf=peripheral blood film, M/h= microcytic hypochromic, and AOR= adjusted odds ratio.

**Table 3:** Association of malaria parasites with changes in maternal haematological parameters.

Malaria parasites						
Variable	OR	CI	P-value	AOR	CI	P-value
Hb level						
<11.5gm/dl	3.451	2.90-5.355	0.001	2.545	2.249-2.879	0.001
**Pbf**						
M/h	3.575	2.784-5.429	0.001	3.011	2.321-6.032	0.001
**Eosinophil**						
>6% Raised	2.271	2.150-2.840	0.001	0.4457	0.310-0.675	0.001

**∗**95% CI=confidence interval and OR= odds ratio; P<0.05 was considered significant.

**∗**Hb= haemoglobin, Pbf=peripheral blood film, M/h= microcytic hypochromic, and AOR= adjusted odds ratio.

**Table 4:** Association of geohelminths and malaria coinfection with changes in maternal haematological parameters.

Geohelminths and Malaria parasites co-infection						
Variable	OR	CI	P-value	AOR	CI	P-value
Hb level						
<11.5gm/dl	6.921	5.486-8.731	0.001	4.137	2.088-8.195	0.001
**Pbf**						
M/h	5.975	4.568-10.321	0.001	5.412	4.913-732	0.001
**Eosinophil**						
>6% Raised	5.558	4.549-6.791	0.001	2.586	1.896-3.967	0.001

**∗**95% CI=confidence interval and OR= odds ratio; P<0.05 was considered significant.

**∗**Hb= haemoglobin, Pbf=peripheral blood film, M/h= microcytic hypochromic, and AOR= adjusted odds ratio.

**Table 5:** Analysis risk factors for adverse neonatal outcomes in single infections.

**Variable**	**Geohelminths alone**		**Malaria parasites alone**	
	Infected	Not infected	Infected	Not infected
**Gestation period**				
>37 weeks Term delivery	160(86.5%)	538(95.2%)	131(88.7%)	567(96.4%)
<37 weeks preterm delivery	18(9.7%)	23(4.1%)	26(16.0%)	15(2.6%)
< 28 weeks abortion	7(3.8%)	4(0.7)	5(3%)	6(1.0)
Chi-square	18.296		49.334	
P-value	0.001		0.001	
**Neonatal condition at birth**				
Live birth	174(94.1%)	553(97.8%)	154(95.1%)	573(97.5%)
Fresh still birth	2(1.1%)	6(1.1%)	2(1.2%)	6(1.0%)
Macerated still birth	9(4.8)	6(1.1%)	6(3.7%)	9(1.5%)
Chi-square	10.288		3.128	
P-value	0.006		0.209	
**Birth weight**				
>2500g Normal birth	158(85.4%)	532(94.2%)	126(77.8%)	564(95.9%)
<2500g Low birth eight	27(14.6%)	33(5.8%)	36(22.0%)	24(4.1%)
Chi-square	14.511		56.788	
P-value	0.001		0.001	
**APGAR score**				
10 Normal	153(82.8%)	531(94%)	130(80.3%)	554(94.2%)
7-9 High chances of survival	18(9.7%)	15(2.7%)	17(10.5%)	16(2.7%)
<6 Low chances of survival	3(1.6%)	7(1.2%)	7(4.3%)	3(0.5%)
0 Dead	11(5.9%)	12(2.1%)	8(4.9%)	15(2.6%)
Chi-square	24.590		36.351	
P-value	0.001		0.001	

**∗**P<0.05 was considered significant.

**Table 6:** Analysis of risk factors for adverse neonatal outcomes in coinfections.

**Variable**	** Co-infection**		
	P-value	OR	95% CI
**Gestation period**			
< 37 weeks Preterm delivery	0.006	6.896	1.755-27.101
< 28 weeks Abortion	0.897	0.906	0.205-4.012
**Neonatal condition at birth**			
Fresh still birth	0.048	3.701	1.008-13.579
Macerated still birth	0.750	0.750	0.098-5.768
**Birth weight**			
Low birth weight	0.001	4.186	3.O94-8.369
**APGAR**			
<6 Low chances of survival	0.001	5.310	1.860-15.162
7-9 High chances of survival	0.478	0.639	0.185-2.204
Dead	0.911	1.111	0.177-6.990

**∗**95% CI=confidence interval and OR= odds ratio; P<0.05 was considered significant.

## Data Availability

The data used to support the findings of this study are available from the corresponding author upon request.
